# Seroprevalence of SARS-CoV-2 antibodies and knowledge, attitude and practice toward COVID-19 in the Republic of Srpska-Bosnia & Herzegovina: A population-based study

**DOI:** 10.1371/journal.pone.0262738

**Published:** 2022-01-28

**Authors:** Biljana Mijović, Srdjan Mašić, Miroslav Petković, Darija Knežević, Jela Aćimović, Jelena Djaković-Dević, Dragana Puhalo-Sladoje, Branislav Zeljković, Dragan Spaić, Dejan Bokonjić, Ranko Škrbić

**Affiliations:** 1 Faculty of Medicine, Department of Primary Health Care and Public Health, University of East Sarajevo, Foča, the Republic of Srpska, Bosnia and Herzegovina; 2 Public Health Institute of the Republic of Srpska, Banja Luka, the Republic of Srpska, Bosnia and Hercegovina; 3 Faculty of Medicine, University of Banja Luka, Centre for Biomedical Research, Banja Luka, the Republic of Srpska, Bosnia and Herzegovina; 4 Faculty of Medicine, University of East Sarajevo, Centre for Biomedical Research, Foca, the Republic of Srpska, Bosnia and Herzegovina; 5 Faculty of Medicine, Department of Paediatrics, University of East Sarajevo, Foča, the Republic of Srpska, Bosnia and Herzegovina; Public Health England, UNITED KINGDOM

## Abstract

The aim of the study was to assess the seroprevalence of SARS-CoV-2 in the Republic of Srpska, Bosnia and Herzegovina, and to analyse the knowledge, attitudes and practices of the population toward COVID-19. This population-based study was conducted in a group of 1,855 randomly selected individuals from all municipalities from 1 December 2020 to 15 January 2021. All individuals were asked to sign a consent form and to fill in a questionnaire, following which a blood samples were collected. Total anti-SARS-CoV-2 antibodies were determined in serum specimens using the total Ab ELISA assay. The overall seroprevalence rate was 40.3%. Subjects aged <65 years were 2.06 times more likely to be seropositive than those aged ≥65, and 30% of seropositive individuals presented no COVID-19 symptoms. The household members of seropositive individuals were 2.24 times more likely to develop COVID-19 symptoms than the household members of seronegative individuals. More than 95% of respondents believe that preventive measures are very important to control the infection transmission. Majority of respondents wear the masks properly, maintain the required physical distance whenever possible and wash hands with soap. Nearly 50% of individuals were of the opinion that the vaccine could prevent the infection. This study showed that an overall SARS-CoV-2 seropositivity rate by the middle of January 2021 was very high. Attitudes and practices regarding the COVID-19 indicate that additional efforts should be taken in order to improve the health education with a focus on preventive measures and vaccination.

## Introduction

In the Republic of Srpska, the first case of Coronavirus Disease 19 (COVID-19) was confirmed on March 5, 2020 and the disease had spread rapidly. The first hundred days of the outbreak were characterised with three phases: phase of sporadic cases, clusters of cases and the phase of community transmission. During the summer months, the number of confirmed cases decreased, but the second wave of infection began in early October having peaked in late October. Since early November 2020, the number of confirmed cases showed steady downward trends, but in early March 2021 there was a new increase in the number of cases, which corresponded to the third wave of COVID-19. As at 2 March 2021, a total of 43,787 cases of SARS-CoV-2 were confirmed, with 2,264 deaths [1].

Due to rapid increase in the number of individuals with COVID-19 symptoms that exceeded capacities of laboratories, it was not possible to confirm all cases by reverse transcription polymerase chain reaction (RT-PCR). In addition, as the pandemic evolved, the number of asymptomatic cases was expected to increase. According to Oran et al., approximately 40–45% of individuals with confirmed presence of the virus did not develop any symptom of COVID-19 [2]. The standard statistics on the number of confirmed cases and deaths were not sufficient to assess the epidemiological situation [3], giving right to concern that the number of infected and diseased individuals was significantly higher [4].

In order to protect public health, it is very important to estimate the size of the infected population. A number of studies have been conducted to assess the seroprevalence of SARS-CoV-2 in certain populations [5–8]. A study of Korth et al., at the University Hospital of Essen, found an overall IgG seroprevalence among health care workers of 1.6% [9]. A SARS-CoV-2 seroprevalence study in a healthy adult population among blood donors in the Netherlands found that one month after the outbreak, the seroprevalence was 2.7%, with some regional differences. The most affected areas had a seroprevalence of 9.5%, and antibodies were more common in young people (18–30 years) [10]. Oliveira et al. assessed the seroprevalence of anti-SARS-CoV-2 antibodies in outpatients of a hospital in Sao Paulo. The study was conducted on a sample of 439 patients and the prevalence of IgG antibodies was 13.9%. Among them, 32.8% of patients were asymptomatic. The IgG seroprevalence was lower in patients who received a seasonal flu vaccine, which the authors explained by better personal health care or better health care services [11]. Stefanelli et al. found the IgG seroprevalence of 23.1% among residents of Trento province in Italy [12].

The World Health Organisation (WHO) recommends that a population-based serology test should be performed after the peak of an epidemiological wave [13]. The Faculties of Medicine in Foča and Banja Luka, in collaboration with the Institute of Public Health of the Republic of Srpska launched a study to assess the seroprevalence of SARS-CoV-2 antibodies in the Republic of Srpska population. Here we present the first of two rounds of the study. Moreover, the additional aim of the study was to assess the knowledge, attitude and practice of the population regarding prevention and protection measures against COVID-19.

## Methods

### Study design and participants

Serological testing for SARS-CoV-2 antibodies, as well as the epidemiological survey were conducted as a cross-sectional study from 1 December 2020 to 15 January 2021 on a representative sample of the population. The Republic of Srpska has a population of 1,147,902 inhabitants (408,825 households) [14]. The average household has 2.85 members. Ensuing calculations resulted in a sample size of 1,904 individuals across 667 households (the estimated minimum sample size was 1,728). A sample size of 1,728 produces a two-sided 95% confidence interval with a width equal to 0.020 when the sample proportion is 0.100. The sample size was determined specifically for each city/municipality (out of a total of 64 municipalities in the republic), where households were randomly selected from primary health care centres data bases according to number of inhabitants per city (municipality). The total number of included participants was 1,855 (698 households) and the response rate was 97.43%. The number of participants, as well as the households, is greater than the computed minimal sample size for the study. All residents in households were invited to participate. The study did not include persons accommodated in student dormitories, boarding schools for children and youth with disabilities, nursing homes, prisons, monasteries and convents. The selected participants were invited to come to their primary health care centres for a survey and free serological testing. Interviews and blood specimen collection were performed by nurses and laboratory technicians of primary health care centres according to a protocol designed in advance to this study.

### Epidemiological survey

The participants had to answer the questions from a questionnaire related to COVID-19 symptoms, their contacts with suspected or confirmed cases, understanding of disease prevention and attitudes towards COVID-19. The questionnaire consisted of 73 questions divided into five sections: demographic characteristics; COVID-19 symptom data; COVID-19 cases in the household; risk perception of SARS-CoV-2 infection; compliance with the recommended prevention measures.

### Blood specimens

Blood specimens were collected from all individuals as follows: 3 ml from children and 5 ml from adults. The specimens were proceeded through a centrifuge at 1,500 rpm for 10 minutes. Before being dispatched to the laboratory, they were stored in a refrigerator at a temperature of 2–8°C. On the same or following day, the specimens were delivered, in compliance with a cold-chain protocol, to the microbiological laboratories of the medical faculties, depending on the geographical location where the samples had been collected.

### Detection of SARS-CoV-2 antibodies

Serum specimens were tested for the presence of total SARS-CoV-2 antibodies using the WANTAI Total Ab ELISA assay according to the manufacturer’s instructions in a biosafety level 2 laboratory. The test we used detected the total antibodies, including IgA, IgM and IgG on any kind of virus protein. According to the package insertion (WANTAI SARS-CoV-2 Ab ELISA; Version: V. 2020-02US), the antigen used in this assay is the receptor-binding domain (RBD) of SARS-CoV-2 spike protein. Thus, the test detects the total antibodies against receptor binding domain of the spike protein. The measurement was performed using a Euroimmun ELISA Analyzer I-2P (EUROIMMUN Medizinische Labordiagnostika AG, Lübeck, Germany). The WANTAI SARS-CoV-2 Ab is an ELISA assay for the qualitative assessment of total SARS-CoV-2 antibodies in human serum or plasma. The cut-off value set by the manufacturer for a positive test result is ≥1.1. The test has a sensitivity of 94.36% and a specificity of 100%.

### Statistical analysis

Descriptive statistics were calculated for baseline participants’ characteristics, knowledge, attitudes and practices towards COVID-19. Categorical data were presented as absolute numbers with frequencies. Seroprevalence was determined as a proportion of individuals with a positive test result for total antibodies. Baseline differences between groups were analysed using Pearson chi-squared test for categorical variables. Pearson chi-squared test was used to determine the differences in the knowledge, attitudes and practices. Univariante logistic regression was used to examine the association thereof with a positive serological result. Results were expressed calculating relative risk (RR), and their 95% confidence intervals (CI). All tests were two-tailed. The P value of <0.05 was considered as statistically significant. Statistical analysis was done using IBM SPSS Statistics 26 software.

### Ethical statement

The study was approved by the Ethics Committee of the Faculty of Medicine Foča, University of East Sarajevo (Decision number: 01-2-8 dated November 6, 2020). After a detailed explanation of the study protocol, all participants voluntary signed the consent form. Participants who refused to participate were not included in the study, nor were the members of their families.

## Results

The study included 1,855 participants of whom 809 (43.6%) were male and 1,046 (56.4%) were female. The youngest participant was 1 year old, and the oldest one was 88 years old. The distribution of participants according to age groups and gender is presented in Table 1. In the tested population, 747 had a positive test result, and 1,108 had a negative test result; hence, the seroprevalence in the period from1 December 2020 to 15 January was 40.3%. Among women, the seroprevalence was 38.8%, and among men it was 42.3%. Higher seroprevalence values were found in following age groups: 10–19 (40.1%), 20–34 (47.4%), 35–49 (40.5%) and 50–64 (41.2%), while lower value was identified in the oldest population group ≥ 65 (26.6%). In addition, individuals aged<65 were 2.06 times more likely to have a seropositive result compared to individuals aged ≥65 (p ≤ 0.001) (Table 1).

**Table 1:** Distribution and prevalence of seropositive participants according to gender and age groups.

Variables	Number of participants	%	Number of seropositive	Prevalence %	Relative risk ratio (95% CI)	P value
**Gender**						
Male	809	43.6	341	42.2		
Female	1,046	56.4	406	38.8		
	Male vs. Female				1.15(0.96–1.39)	0.132
**Age groups**						
0–9	62	3.4	22	35.5		
10–19	244	13.2	100	41.0		
20–34	314	16.9	149	47.4		
35–49	534	28.8	216	40.5		
50–64	512	27.6	211	41.2		
≥ 65	189	10.1	49	26.0		
	< 65 vs. ≥ 65				2.06 (1.47–2.89)	p<0.001
**Total**	1,855	100	747	40.3		

CI: Confidence interval.

For seropositive samples the total antibody titre ranged from 1.10 to 62.70; the mean was 34.45 and median was 19.86 (SD 26.63). The close contacts with a confirmed SARS-CoV-2 cases were more common among seropositive individuals compared to seronegative individuals (46% vs. 15.9% p≤0.001). Of the seropositive individuals, 523 (70%) presented a COVID-19 symptoms in the period from 4 March 2020 to the testing date, whereas 30% presented no symptoms at all. There was a significant difference in the results between seropositive and seronegative individuals in terms of symptoms, hospitalisation, and health consultation (Table 2).

**Table 2:** Symptoms and signs of COVID-19 in relation to SARS-CoV-2 seropositivity.

Questions/Symptoms	SARS-CoV-2 serology test result					Relative risk (95% CI)	P value
	Positive		Negative				
	n	%	n		%		
Close contact with a person with confirmed SARS-CoV-2							
Yes	303	46.0		148	15.9	184.77*	≤ 0.001
No	240	36.4		611	65.6		
I don’t know	116	17.6		172	18.5		
Fever							
Yes	240	33.1		72	6.6	7.02 (5.27–9.33)	≤ 0.001
No	484	66.9		1019	93.4		
Anosmia / ageusia							
Yes	262	36.0		24	2.2	25.02 (16.24–38.54)	≤ 0.001
No	465	64.0		1,066	97.8		
Chills							
Yes	187	26.0		72	6.6	4.97(3.714–6.65)	≤ 0.001
No	532	74.0		1,018	93.4		
Nausea							
Yes	142	19.9		75	6.9	3.35 (2.48–4.51)	≤ 0.001
No	573	80.1		1,014	93.1		
Myalgia							
Yes	296	41.0		140	12.8	4.71 (3.74–5.94)	≤ 0.001
No	426	59.0		950	87.2		
Sore throat							
Yes	177	24.6		140	12.9	2.20 (1.72–2.82)	≤ 0.001
No	543	75.4		948	87.1		
Cough							
Yes	248	34.2		174	15.9	2.74 (2.19–3.429)	≤ 0.001
No	478	65.8		919	84.1		
Rhinorrhoea							
Yes	208	28.7		221	20.2	1.58 (1.27–1.97)	≤ 0.001
No	517	71.3		872	79.8		
Dyspnoea							
Yes	132	18.4		48	4.4	4.87 (3.44–6.88)	≤ 0.001
No	587	81.6		1,040	95.6		
Chest pain							
Yes	128	17.7		65	6.0	3.37 (2.46–4.62)	≤ 0.001
No	594	82.3		1.018	94.0		
Headache							
Yes	306	42.6		232	21.8	2.66 (2.16–3.27)	≤ 0.001
No	412	57.4		832	78.2		
Vomiting							
Yes	94	5.3		57	5.4	2.68 (1.90–3.79)	≤ 0.001
No	612	86.7		998	94.6		
Abdominal pain							
Yes	94	13.2		59	5.6	2.57 (1.83–3.61)	≤ 0.001
No	617	86.8		997	94.4		
Diarrhoea							
Yes	131	18.5		98	9.3	2.21 (1.67–2.93)	≤ 0.001
No	578	81.5		957	90.7		
Consulted a doctor due to any of the symptoms							
Yes	326	45.7		141	13.3	5.49 (4.36–8.91)	≤ 0.001
No	387	13.3		919	86.7		
Hospitalised due to any of the symptoms							
Yes	43	6.0		5	0.5	13.44 (5.29–34.11)	≤ 0.001
No	671	94		1,049	99.5		

* The values refer to the Chi-square test; CI: Confidence interval; SARS-CoV-2: Severe Acute Respiratory Syndrome Coronavirus.

A total of 550 individuals (29.6%) had at least one member of their household with suspected or confirmed COVID-19, of whom 329 (47.1%) were seropositive individuals and 221 (21.1%) seronegative individuals (p≤0.001). Among the seropositive household members with suspected or confirmed COVID-19 there were 293 individuals (80.3%) with symptoms. The household members of seropositive individuals were 2.6 times more likely to be hospitalised than the household members of seronegative individuals (p = 0.003). Hospitalisation prior to onset of symptoms was not identified as a risk of infection (Table 3).

**Table 3:** COVID 19 symptoms and signs among household members in relation to seropositivity.

Questions and answers	SARS-CoV-2 serology test results				Relative risk (95% CI)	P value
	Positive		Negative			
	N	%	n	%		
Were there any suspected or confirmed COVID-19 cases in your household?						
Yes	329	47.1	221	21.1	3.32 (2.69–4.10)	≤ 0.001
No	369	52.9	825	78.9		
Did any of your household members with COVID-19 have any symptoms?						
Yes	293	80.3	192	64.6	2.24 (1.58–3.18)	≤ 0.001
No	72	19.7	106	35.6		
Were any of your household members with confirmed COVID-19 hospitalised?						
Yes	43	11.5	14	4.7	2.60 (1.39–4.86)	0.003
No	331	88.5	281	96.3		
Were the members of your household in home isolation prior to the onset of symptoms or confirmation of COVID-19?						
Yes	202	54.0	98	33.2	2.36 (1.72–3.23)	≤ 0.001
	172	46.0	197	66.8		
Was the household member hospitalised prior to the onset of symptoms for any reason?						
Yes	10	2.8	3	1.1	2.60 (0.71–9.56)	0.149
No	353	97.2	276	98.9		

COVID-19: Coronavirus disease 19; CI: Confidence interval; SARS-CoV-2: Severe Acute Respiratory Syndrome Coronavirus.

Most of the respondents, i.e. 633 seropositive (87.6%) and 947 seronegative (87.6%), considered the COVID-19 to be a very serious disease. As many as 524 seropositive respondents (72.5%) and 734 seronegative respondents (67.9%) believed it was quite likely that they themselves or persons in their immediate surroundings would contract the COVID-19. Most of the respondents thought they would not be able to perform everyday activities if they contracted the COVID-19, i.e. 530 seropositive (73.5%) and 730 seronegative (67.9%), and this difference was statistically significant. Only half of the respondents believed the disease could be prevented with a vaccine (48.9% of the seropositive and 51.3% of the seronegative) (Table 4).

**Table 4:** People’s knowledge, attitude and practice related to COVID-19.

Questions and answers	SARS-CoV-2 serology test results				X^2^/F	P value
	Positive		Negative			
	N	%	n	%		
COVID-19 infection is very serious disease.						
I disagree.	25	3.5	27	2.5	1.76	0.415
I am indifferent	65	9.0	107	9.9		
I agree.	633	87.6	947	87.6		
It is highly likely that I or persons around me will be infected with COVID-19.						
I disagree.	57	7.9	106	9.8	4.51	0.105
I am indifferent.	142	19.6	241	22.3		
I agree.	524	72.5	734	67.9		
If I am infected with COVID-19, I will be able to go about my everyday routines as usual.						
I disagree.	530	73.5	730	67.9	8.36	0.015
I am indifferent.	110	15.3	220	20.5		
I agree.	81	11.2	125	11.6		
There aren’t many infected people in our country.						
I disagree.	493	68.6	738	69.4	0.20	0.904
I am indifferent.	102	14.2	151	14.2		
I agree.	124	17.2	175	16.4		
This disease could be prevented with a vaccine.						
I disagree.	109	16.2	138	14.1	1.66	0.437
I am indifferent.	234	34.9	338	34.6		
I agree.	328	48.9	501	51.3		
I am worried about COVID-19.						
Yes	335	40.8	486	59.2	8.16	≤ 0.001
No	378	50.7	368	49.3		
Do you expect a financial crisis due to the COVID-19?						
Unlikely	98	13.7	163	15.2	2.10	0.350
Somewhat likely	231	32.2	367	34.1		
Highly likely	388	54.1	545	50.7		
How likely is non-compliance with measures aimed at curbing the spread of the infection?						
Unlikely	78	11.01	103	9.8	2.03	0.361
Somewhat likely	226	31.9	314	29.8		
Highly likely	404	57.0	636	60.4		

SARS-CoV-2: Severe Acute Respiratory Syndrome Coronavirus;X^2^/F: Chi-square Test/Fisher Exact Test; CI: Confidence interval.

Regarding the compliance with preventive measures, most of the respondents (96.1% of seropositive and 97.4% of seronegative) believed that the measures were very important, whereas only 2.6% of seronegative respondents and 3.9% of seropositive respondents found them totally unnecessary; these difference was significant (p = 0.007). Most of the respondents in both groups wore a mask outdoors and indoors. As much as 86.3% of seronegative respondents and 84.5% of seropositive respondents said they wore the mask covering the nose and mouth. There was no difference in the number of seropositive respondents in relation to wearing a fabric or surgical mask (p = 0.068). Mask wearing was found more difficult by seropositive respondents (36.8%) compared to seronegative respondents (27.6%), and that difference was statistically significant (p = 0.002). The majority of respondents said that they maintain the required physical distance whenever possible and wash hands with soap (Table 5).

**Table 5:** Compliance with recommended preventive measures.

Questions	SARS-CoV-2 serology test results				Relative risk(95% CI)	P value
	Positive		Negative			
	n	%	n	%		
I find that the preventive measures introduced to stop COVID-19 transmission are:						
Quite unnecessary	28	3.9	28	2.6	1.26 (1.06–1.49)	0.007
Very important	699	96.1	1,058	97.4		
I wear a mask outdoors.						
Yes	472	64.7	741	67.9	0.91 (0.82–1.01)	0.802
No	258	35.3	351	32.1		
I wear a mask indoors.						
Yes	569	77.6	870	79.6	0.92 (0.82–1.05)	0.243
No	164	22.4	223	20.4		
I wear a mask so that it completely covers my nose and mouth.						
Yes	617	84.5	940	86.3	0.92 (0.80–1.06)	0.280
No	24	15.5	33	13.7		
I usually use:						
Fabric (cloth) masks	304	41.7	407	37.4	1.19 (0.98–1.45)	0.068
Surgical (medical) masks	425	58.3	680	62.6		
Mask wearing is uncomfortable.						
Yes	269	36.8	301	27.6	1.19 (1.07–1.33)	0.002
No	461	63.2	790	72.4		
I maintain the required physical distance.						
Yes	499	68.3	742	68.0	1.01 (0.91–1.12)	0.927
No	232	31.7	349	32.0		
I wash hands with soap and water rubbing them for at least 20 seconds.						
Yes	654	89.5	994	91.2	0.91 (0.77–1.07)	0.249
No	77	10.5	96	8.8		

## Discussion

The results of this study showed that SARS-CoV-2 seroprevalence in the Republic of Srpska by mid-January 2021 was 40.3%, which is higher than the values found by other studies published so far. There were no differences in seroprevalence by gender in the study population. Pollan et al. conducted a SARS-CoV-2 national seroprevalence study in Spain. The point-of-care tests detected seroprevalence of 5.0%, while serology tests detected seroprevalence of 4.6% [15]. Studies conducted in Geneva in a group of 2,766 participants older than five years from 1,339 households found that as of week one the seroprevalence was 4.8%, and 8.5%, 10.9%, 6.6% and 10.8% in following weeks. The study showed the lowest infection rates in age groups 5–9 age and ≥ 65, and that the majority of Geneva’s population was uninfected [8]. These studies were conducted well before the onset of the second wave, whereas our study was conducted more than two months after the onset of the second wave of the epidemic, hence the higher seroprevalence. The seroprevalence varied with age groups, with the lowest prevalence in the age group ≥65 (26%) and the highest prevalence in the age group 20–34 (47.4%). Individuals aged ≥65 were 2.06 times less often to have a seropositive result than other individuals, which can be explained by the social isolation and better compliance with recommended preventive measures. Individuals aged ≥65 were advised from health professionals to follow very strict rules related to quarantine and movement restriction. Moreover, the participants aged ≥65 largely respected most of the preventive measures such as “wearing a mask outdoors”. Also, participants ≥65 years respected some others preventive measures to a greater extent than youngers: “maintain the required social distance”, “physical distance”, “washing hands with soap and water rubbing them for at least 20 seconds”, and “disinfection of surface at home”. In addition, the immune response may be weaker at this age. The Spanish and Geneva studies found the highest seroprevalence in the age group ≥65 (6.0%) (14,8). Lai et al. found that in almost all studies they analysed the children were at a lower risk of infection than other age groups [16]. Children may have a different immune response and, including a less prominent nasal expression of the angiotensin-converting enzyme [17]. However, in our study the seroprevalence among the children age 1–9 and younger adolescents (age 10–19) were 35.55% and 41%, respectively. According to the census of the Republic of Srpska children of age 0 to 9 are taking 9.2% of the total population and in our sample only 3.4%. Therefore, our sample might not be the best representative of the children population. Also, it is possible that there was grouping of seropositive participants per household which already had confirmed cases of COVID-19 infection.

The study showed that 46% of seropositive and 15.9% of seronegative respondents said they had contact with a person with confirmed SARS-CoV-2. Therefore, since it is difficult to determine the actual number of COVID-19 patients, a seroprevalence study for SARS-CoV-2 can contribute to estimate the actual size of infected population. In this study, 70% of seropositive individuals presented at least one symptom of the COVID-19, while others (30%) had no symptoms at all. In addition, 80% of household members of seropositive persons, with suspected or confirmed infection, presented at least one of COVID-19 symptoms. In Spanish study, the asymptomatic cases accounted for 21–35.8% of all SARS-CoV-2 infections confirmed with antibody tests [15]. In the presented study, 53.8% of symptomatic individuals had a positive serology test for SARS-CoV-2 antibodies, whereas the Spanish study found only 15.3–19.3% symptomatic cases [15]. They also found that significant proportion of suspected cases were not caused by the SARS-CoV-2 virus. The S protein and nucleoprotein are known to have less than 30% similarity to endemic beta-coronaviruses, hence the cross-reaction cannot be ruled out [18].

Detailed understanding of the seroprevalence of asymptomatic COVID-19 cases and the clinical characteristics of mild COVID-19 symptoms are essential for effective control of the COVID-19 pandemic. As much as 550 household members had a suspected or confirmed infection, of whom 329 (47.1%) had a positive and 221 (21.1%) had negative serology test results. The COVID-19 had high transmission rates across the households. The high rates may be explained with close, trans-generational contact, which is common in our households. The COVID-19 transmission in community was contained by imposing home or hospital isolation of suspected or confirmed cases. The republic inspectorate issued procedural decisions ordering home isolation of individuals with a positive RT-PCR test result. Although the current recommendations require that home isolation should be in place not only for confirmed, but also for suspected cases, it was not always implemented in practice. Our study has found that the participants did not always report the symptoms to the health service. Thus, 326 of seropositive respondents (45.7%) and 141 (13.3%) of seronegative respondents consulted a doctor.

Perception of the risk of infection caused by SARS-CoV-2 can be highly relevant for understanding the actual onset of the infection. In our study, a set of parameters of risk perception were examined, and risk perception was found to be quite similar in all areas covered. A survey of knowledge, risk perception and strategies for handling the pandemic, conducted by Führer et al. in Germany, found that 60% of the respondents reported fears for the well-being of family members [19]. In the same survey, 79% of the respondents reported concerns regarding adverse economic impacts, whereas in our survey 52% of the respondents said the pandemic was likely to cause a financial crisis.

The non-compliance with epidemic measures contributed to the spread of the COVID-19. In our survey, 60.4% of seronegative respondents and 57.1% of seropositive respondents thought that non-compliance was very likely. Prevention was understood as the best way to manage the COVID-19 pandemic. As regards preventive measures, the survey found that over 95% of the respondents believed that preventive measures were important. In the German survey, most of the respondents regarded the government-mandated safety measures as predominantly reasonable and appropriate [19]. The acceptance of COVID-19 prevention measures by the population is the basis of pandemic control, and adherence to the measures is influenced by their knowledge, attitudes and practices. Our study found that almost the same proportion of participants wore a mask when outdoors (64.7% of seropositive vs. 67.9% of seronegative), and indoors (77.6% of seropositive vs. 79.6% of seronegative), with no significant difference. Most of the respondents wore the mask correctly, so that it covered both, the nose and the mouth. No difference was found in the frequency of positive results with regard to wearing a fabric or surgical masks.

A review of the literature by Dehaghi et al. [20], which included various databases before 30 April 2020, concluded that one in five studies found no difference between surgical and cotton masks in the prevention of COVID-19. Another two studies indicated the importance of wearing a surgical mask or N95 mask by health workers, and yet another two studies highlighted the use of any type of face mask by the public [20]. The results of our study concerning mask wearing are significantly lower than in study by Zhong et al. who found that almost all respondents (98%) wore masks [21].

Regarding the maintenance a physical distance in public spaces, 68% of respondents in our study adhered to this measure. This difference could be explained by culture difference knowing that Asian nations wear masks more often than Europeans. The study conducted by Führer et al. found that 79% of the respondents adhered to protective measures, such as reducing social contacts and maintaining the recommended physical distance in public spaces [19]. Chu et al. conducted a systematic review and meta-analysis in which they found that transmission of viruses was lower with physical distancing of 1 meter or more, compared with a distance of less than 1 meter, and protection was increased as distance was lengthened. It was also found that mask wearing could result in a large reduction in risk of infection with stronger associations with N95 masks compared with disposable surgical masks or cotton masks. Eye protection also was associated with lower infection rates [22]. Two online studies conducted in United States (US) and United Kingdom (UK) found that study respondents were generally well acquainted with the main mode of transmission of the disease and common symptoms, but significant number had misconceptions about how to prevent infection and how to behave. Thus, 37.8% of the US participants and 29.7% of the UK participants considered mask wearing ‘highly effective’ against the COVID-19 [23]. Mohamad et al. were among the first to conduct an online survey of knowledge, attitudes and practices concerning COVID-19 prevention in Malaysia among 4,850 respondents. Most of the respondents avoided crowds (83.4%) and practiced proper hand hygiene (87.8%). However, mask wearing was less prevalent (51.2%) [24]. The results of our study are very similar since 90% of respondents confirmed they practiced hand washing properly.

The key advantage of the presented study is the random selection of households from the register of patients of family medicine teams at the primary health care centres across the Republic of Srpska, which enabled us to identify representative sample size. Another advantage is that the study covered all population groups. However, the random selection of households and the inclusion of young children meant more time was needed to complete the study.

Our study showed that just 50% of individuals believed that the vaccine could prevent the COVID-19 infection. This is rather surprising having in mind that people in this country historically have very positive attitude toward vaccination program, in general. The possible explanation for this distrust in newly developed COVID-19 vaccines could be found in very aggressive anti-vaccination campaigns and rapid dissemination of unproven information on social media. It is time for stakeholders, health professionals, policy makers and researchers for more pro-active approach to improve the acceptance rate of COVID-19 vaccine in order to minimize the morbidity and mortality related to COVID-19 pandemic. The population has to be aware regarding the importance, safety and efficacy of COVID-19 vaccine.

There are several limitations of the study. At the first place, the study was design to measure the total antibodies, meaning that individuals with recent acute infection were also included in the study. Second, the study did not include cellular immunity, which has a significant role against COVID-19 re-infection. Third, there was no confirmed circulation of influenza virus at the time of this study, and some of the symptoms described by the participants could be attributed to influenza virus as well. Fourth, the children population was not adequately represented in the study; they number was lower than initially calculated since the parents in some cases refused the blood samples to be taken from their kids. The clustering method was not used in this study, but the sample size was carefully planned in order to represent the actual population of the Republic of Srpska, including the participants from both, rural and urban areas.

## Conclusions

Over 40% of the population was found to be seropositive as by the middle of January 2021, and 30% of those cases were asymptomatic. Younger subjects were 2.06 times more likely to be seropositive than those aged ≥65. The household members of seropositive individuals were 2.24 times more likely to develop COVID-19. More than 95% of respondents believe that preventive measures introduced to stop COVID-19 transmission are very important. Majority of respondents wear the masks outdoors and indoors, maintain the required physical distance whenever possible and wash hands properly. Nearly 50% of individuals believed that the vaccine could prevent the infection. The study enabled us to assess the patterns of infection in different age groups, the ratio of asymptomatic infections and other characteristics of the epidemic, and to direct preventive measures in a more efficient way. The study has certainly produced important findings and can contribute to better understanding of COVID-19 disease, which may be useful for a future response to a new epidemic waves.

## Supporting information

S1 File COVID-19 database. (XLSX)

S2 File Questionnaire. (PDF)
