# Supplementary material for: Seroprevalence of SARS-CoV-2 antibodies and knowledge, attitude and practice toward COVID-19 in the Republic of Srpska-Bosnia & Herzegovina: A population-based study
Source: PLoS One. 2022 Jan 28;17(1):e0262738. doi: 10.1371/journal.pone.0262738 (PMC8797215; doi:10.1371/journal.pone.0262738)
Supplement: S2 File — (PDF) [file pone.0262738.s002.pdf]

PROTOCOL FOR THE SEROEPIDEMIOLOGICAL SURVEY OF THE POPULATION ON THE COVID-19 VIRAL INFECTION

FORM 1: EPIDEMIOLOGICAL SURVEY

Single protocol reference number	

Dear respondent,
Thank you for accepting to participate in this national survey, which aims to determine the level of our population's resistance to the infection caused by a novel coronavirus (COVID-19).
The information collected through this questionnaire will be used solely for the purpose of this survey and its safety is guaranteed.

1. INTEVIEWER'S PARTICULARS	
Interviewer's first and family name	
Institution that has engaged the interviewer	
Institution's telephone number	
Cell phone number	
E-mail	
Date of the interview with the respondent	___ / ___/___ (day/month/year)

2. RESPONDENT'S PARTICULARS	
First name	
Family name	
Sex	☐ Male ☐ Female
Date of birth	___/___/___ (day/month/year)
Telephone number	
Age	
E-mail	
Residence	
Occupation	

**3. SYMPTOMS**	
Since 1 March 2020 to date, have you had any of the following symptoms:	
Body temperature ≥38°C	☐ YES ☐ NO
Loss of smell/loss of taste	☐ YES ☐ NO
Fever	☐ YES ☐ NO
Fatigue	☐ YES ☐ NO
Muscle pain	☐ YES ☐ NO

Sore throat	◻ YES ◻ NO
Cough	◻ YES ◻ NO
Runny nose	◻ YES ◻ NO
Breathing difficulties / shortness of breath	◻ YES ◻ NO
Wheezing	◻ YES ◻ NO
Chest pain	◻ YES ◻ NO
Other respiratory symptoms	◻ YES ◻ NO<br>*If the answer is YES, please specify:*____________________
Headache	◻ YES ◻ NO
Nausea / vomiting	◻ YES ◻ NO
Abdominal pain	◻ YES ◻ NO
Diarrhoea	◻ YES ◻ NO
Did you consult a doctor with regard to any of the above symptoms?	◻ YES ◻ NO
Did you ask for a sick leave from work or school due to any of the above symptoms?	◻ YES ◻ NO
Were you hospitalised due to any of the above symptoms?	◻ YES ◻ NO

**4. INFORMATION ON THE COMPLIANCE WITH THE RECOMMENDED PREVENTATIVE MEASURES**	
I deem the preventative measures to contain the transmission of the COVID-19 viral disease:	◻ VERY IMPORTANT ◻ USEFUL<br>◻ QUITE UNNECESSARY
I comply with the recommended measures as follows:	
I wear a face mask when outdoors	◻ YES, ALWAYS ◻ NO, NEVER ◻ OCCASIONALLY
I wear a face mask when indoors	◻ YES, ALWAYS ◻ NO, NEVER ◻ OCCASIONALLY
I wear a face mask in a way that fully covers my nose and mouth	◻ YES, ALWAYS ◻ NO, NEVER ◻ OCCASIONALLY
I wear a single face mask for more than three hours	◻ YES, ALWAYS ◻ NO, NEVER ◻ OCCASIONALLY
I generally use:	◻ COTTON (FIBER) MASKS<br>◻ SURGICAL (MEDICAL) MASKS
The use a face mask increased my feeling of security and safety from the COVID-19 viral infection	◻ YES, ALWAYS ◻ NO, NEVER ◻ OCCASIONALLY
The use of a mask is inconvenient	◻ YES, ALWAYS ◻ NO, NEVER ◻ OCCASIONALLY
Social distancing (avoiding to leave home unless necessary to go to work, a grocery store, a pharmacy)	◻ YES, ALWAYS ◻ NO, NEVER ◻ OCCASIONALLY
Physical distancing (maintaining a distance of at least 2 m from others)	◻ YES, ALWAYS ◻ NO, NEVER ◻ OCCASIONALLY
Washing hands with water and soap and rubbing them for at least 20 seconds	◻ YES, ALWAYS ◻ NO, NEVER ◻ OCCASIONALLY
Disinfecting surfaces at home	◻ YES, ALWAYS ◻ NO, NEVER ◻ OCCASIONALLY
Disinfecting surfaces at work	◻ YES, ALWAYS ◻ NO, NEVER ◻ OCCASIONALLY

5. THE COVID-19 RISK PERCEPTION			
**A. To which extent do you agree with the following statements? (specify by x, one for each row)**			
	I disagree	I am indifferent	I agree
The COVID-19 infection is a very serious disease.			
It is highly likely that I or persons from my close environment will get the COVID-19 infection.			
I am more likely to get the COVID-19 infection than other persons.			
If I get the COVID 19 infection, I would be able to carry on with everyday life activities normally.			
Not many people have got the COVID-19 infection in our country.			
The vaccine could prevent this disease.			
**B. I have been concerned about the COVID-19 infection disease:**			
For the past week	◻ Yes	◻ NO	
For the past month	◻ Yes	◻ NO	
For the past two months	◻ Yes	◻ NO	
For several months	◻ Yes	◻ NO	
**C. As the main source of information about COVID-19 I use:**			
Social media	◻ Yes	◻ NO	
Newspapers, TV, radio	◻ Yes	◻ NO	
Official Government's web site or press statements of officials	◻ Yes	◻ NO	
World Health Organisation	◻ Yes	◻ NO	
Other (specify)	◻ Yes	◻ NO	
**D. How likely is it for you to be affected by any of the following in the next year? (specify the likelihood by "x" in each row)**			
	Absolutely unlikely	Somewhat likely	Very likely
Financial crisis			
Criminal			
COVID-19 infection			
Corruption			
Indecent behaviour of others			

6. THE COVID-19 AMONG HOUSEHOLD MEMBERS		
**Since 1 March 2020 to date:**		
Has any member of your familyhad suspected or confirmed COVID-19?	◻ YES	◻ NO
If the answer is YES:		
How many family members have had suspected or confirmed COVID-19?	________	
Did the family members with a confirmed / suspected COVID-19 viral infection have any symptoms of the said disease?	◻ YES	◻ NO
Did the family members with a confirmed / suspected COVID-19 viral infection have to be hospitalised due to the severity of the symptoms?	◻ YES	◻ NO
Were the family members with a confirmed COVID-19 infection accommodated in a Republic-level facility for SARS-COV-2 positive persons?	◻ YES	◻ NO
Prior the onset of symptoms (or laboratory confirmation using PCR test), were the said family members in home isolation?	◻ YES	◻ NO
Prior to the onset of symptoms (or laboratory confirmation using PCR test), were the family members accommodated in a quarantine facility at a border crossing	◻ YES	◻ NO

point or a quarantine facility in the local community?	
Prior to the onset of symptoms (or laboratory confirmation using PCR test), were the family members in contact with a person with a suspected or confirmed COVID-19 viral infection?	◻ YES ◻ NO
Prior to the onset of symptoms (or laboratory confirmation using PCR test), were they hospitalised in a health facility?	◻ YES ◻ NO
**Suspected case - a person with acute respiratory infection symptoms (fever plus at least 1 sign (cough / difficult breathing / shortness of breath) and positive epidemiological history (travelled to a country affected by the virus or in contact with a positive case within 14 days after the onset of symptoms) but without laboratory confirmation of the infection.**<br>**Confirmed case - a person with laboratory confirmation of the infection with the SARS-COV-2 virus irrespective of clinical signs and disease symptoms**	

PROTOCOL FOR THE SEROEPIDEMIOLOGICAL SURVEY OF THE POPULATION ON THE COVID-19 VIRAL INFECTION

FORM 2: LABORATORY RESULTS

This table must be filled in for each collected sample in accordance with the study design and pre-determined sample collection schedule.

Serological testing method and results (a new table should be used for each collected sample)	
Laboratory ID number	
Date of sample collection	___/___/___
Date of sample receipt by laboratory	___/___/___
Sample type	☐ Serum
Serological test results	☐ Positive<br>☐ Negative
COVID-19 viral titre	
Date of receipt of test results	___/___/___
Has the sample been sent to another laboratory for the confirmation of results?	☐ Yes ☐ No<br>Date ___/___/___
Results of the test conducted by another laboratory	☐ Positive<br>☐ Negative

PROTOCOL FOR THE SEROEPIDEMIOLOGICAL SURVEY OF THE POPULATION ON THE COVID-19 VIRAL INFECTION

FORM 3: INFORMED CONSENT

Dear sir / madam,

The above survey conducted by the School of Medicine in Foča, the RepublikaSrpska Public Health Institute and the School of Medicine in Banja Luka aims to understand the level of exposure of the RepublikaSrpska population to the COVID-19 disease.

The COVID 19 disease has occurred in the entire world as a pandemic and is caused by a novel virus called SAR CoV-2. Since this is a new disease, there are many uncertainties that need exploring so that we should be able to fight it in a better way. Among other things, it is not known to what extent the RepublikaSrpska population has been exposed to this infection, how long the antibodies in our system will work and whether they will have a protective role in the next encounter with the infection.

Our survey will look into the seroprevalence of the COVID 19 disease in the RepublikaSrpska, which will help us understand the our country's resistance to the infection caused by the coronavirus and plan future public health activities concerning the response to a new epidemic wave.

The survey will be conducted by trained health professionals. Blood samples from patients and epidemiological information will be collected from patients. The blood samples will be processed at the RepublikaSrpska Public Health Institute.

This survey is anonymous and all collected data will be used solely for research purposes.

With my signature I confirm that I have received all necessary information on the goals and benefits of this survey and agree to participate in this survey on a voluntary basis.

Signature

PROTOCOL FOR THE SEROEPIDEMIOLOGICAL SURVEY OF THE POPULATION ON THE COVID-19 VIRAL INFECTION

FORM 3: INFORMED CONSENT

Dear sir / madam,

The above survey conducted by the School of Medicine in Foča, the RepublikaSrpska Public Health Institute and the School of Medicine in Banja Luka aims to understand the level of exposure of the RepublikaSrpska population to the COVID-19 disease.

The COVID 19 disease has occurred in the entire world as a pandemic and is caused by a novel virus called SAR CoV-2. Since this is a new disease, there are many uncertainties that need exploring so that we should be able to fight it in a better way. Among other things, it is not known to what extent the RepublikaSrpska population has been exposed to this infection, how long the antibodies in our system will work and whether they will have a protective role in the next encounter with the infection.

Our survey will look into the seroprevalence of the COVID 19 disease in the RepublikaSrpska, which will help us understand the our country's resistance to the infection caused by the coronavirus and plan future public health activities concerning the response to a new epidemic wave.

The survey will be conducted by trained health professionals. Blood samples from patients and epidemiological information will be collected from patients. The blood samples will be processed at the RepublikaSrpska Public Health Institute.

This survey is anonymous and all collected data will be used solely for research purposes.

With my signature I confirm that I have received all necessary information on the goals and benefits of this survey and agree to participate in this survey on a voluntary basis.

Signature
